# Gray's Time-Varying Coefficients Model for Posttransplant Survival of Pediatric Liver Transplant Recipients with a Diagnosis of Cancer

**DOI:** 10.1155/2013/719389

**Published:** 2013-05-12

**Authors:** Yi Ren, Chung-Chou H. Chang, Gabriel L. Zenarosa, Heather E. Tomko, Drew Michael S. Donnell, Hyung-joo Kang, Mark S. Roberts, Cindy L. Bryce

**Affiliations:** ^1^Department of Biostatistics, Graduate School of Public Health, University of Pittsburgh, Pittsburgh, PA 15261, USA; ^2^Department of Medicine, School of Medicine, University of Pittsburgh, Pittsburgh, PA 15261, USA; ^3^Department of Clinical and Translational Science, School of Medicine, University of Pittsburgh, Pittsburgh, PA 15261, USA; ^4^Department of Industrial Engineering, Swanson School of Engineering, University of Pittsburgh, Pittsburgh, PA 15261, USA; ^5^Department of Health Policy and Management, Graduate School of Public Health, University of Pittsburgh, Pittsburgh, PA 15261, USA; ^6^Health Geography Lab/Biostatistics Research Group, Division of Preventive and Behavioral Medicine, University of Massachusetts Medical School, Worcester, MA 01605, USA

## Abstract

Transplantation is often the only viable treatment for pediatric patients with end-stage liver disease. Making well-informed decisions on when to proceed with transplantation requires accurate predictors of transplant survival. The standard Cox proportional hazards (PH) model assumes that covariate effects are time-invariant on right-censored failure time; however, this assumption may not always hold. Gray's piecewise constant time-varying coefficients (PC-TVC) model offers greater flexibility to capture the temporal changes of covariate effects without losing the mathematical simplicity of Cox PH model. In the present work, we examined the Cox PH and Gray PC-TVC models on the posttransplant survival analysis of 288 pediatric liver transplant patients diagnosed with cancer. We obtained potential predictors through univariable (*P* < 0.15) and multivariable models with forward selection (*P* < 0.05) for the Cox PH and Gray PC-TVC models, which coincide. While the Cox PH model provided reasonable average results in estimating covariate effects on posttransplant survival, the Gray model using piecewise constant penalized splines showed more details of how those effects change over time.

## 1. Introduction

Transplantation is often the only viable treatment for children with end-stage liver disease [1], but the shortage of donor livers means that not every child on the waiting list can receive a transplant. Since 2002, prioritization on the waiting list is determined by the model for end-stage liver disease (MELD)/pediatric end-stage liver disease (PELD) severity score, which allocates organs to the sickest individuals first [2]. However, survival outcomes still vary, suggesting that long-term survival is affected by factors other than illness severity at time of transplant.

 For example, posttransplant survival is particularly poor for certain diagnoses such as primary liver malignancies (cancer). Among children transplanted during the MELD/PELD era, disease-specific Kaplan-Meier survival plots indicate that transplant recipients with cancer had significantly lower posttransplant survival rates than those with other diseases (logrank test *P* < 0.001). 

 We used this subgroup of transplant recipients to compare two alternative methods for estimating posttransplant survival and its significant covariates. Traditionally, survival models have been developed using Cox proportional Hazards (PH) models [3], but some diseases do not adhere to the basic assumption of proportional hazards, implying that the covariate effects are not constant over time. In such cases, an alternative survival model that accounts for varying covariate effects must be used, and we chose Gray's piecewise constant time-varying coefficient (PC-TVC) model [4]. The objective of the paper is to demonstrate that Gray PC-TVC model can provide more flexibility in capturing the temporal dynamics of covariate effects during posttransplant period.

## 2. Methods

### 2.1. OPTN Data

The organ procurement and transplantation network (OPTN) maintains national-level data on all transplant candidates. We obtained a standard transplant analysis and research (STAR) file and restricted the file to 76,233 adult and pediatric liver transplant candidates listed since the MELD/PELD scoring system was first implemented (02/27/2002 through 06/25/2010). We then removed adults age of 18 years or older (*n* = 70,506). Of the remaining candidates, we excluded 2,252 patients who never received a transplant, who received a multiorgan transplant, or whose transplantation date occurred before listing, leaving a pediatric cohort of 3,471 liver transplant recipients for the posttransplant patient survival analysis. We then selected 288 (8.3%) pediatric recipients from the cohort with a diagnosis of cancer at time of transplant as the final cohort. 

### 2.2. Covariates

The following 26 variables are included in our study: recipient age, gender, blood type, African-American ethnicity, or other; donor age, gender, blood type, race/ethnicity, donor type (cadaveric, living); recipient-donor blood type compatibility, transplant year, procurement distance, “exceptional” transplant case (indicating medical concerns that are not fully reflected in the candidate's MELD/PELD score), waiting time, laboratory values (albumin, bilirubin, INR, creatinine) at time of transplant, positive cytomegalovirus (CMV) test, transplant center location (based on 11 geographic regions defined by UNOS), allocation type, presence of ascites, split liver; presence of portal vein thrombosis, on ventilator at time of transplant, and previous abdominal surgery. 

Among 288 children, one recipient had missing values in recipient age, donor age, donor gender, donor type, transplant year, and ventilator use; 18 recipients did not have serum creatinine values (6.25%). Since there is no strong clinical reason to believe that these missing values are related to survival or to other covariates, we treated the missing type as missing completely at random (MCAR) and used complete-case analysis in our original paper. We later performed a sensitivity analysis, treating missing type as missing at random (MAR) and rerunning the multivariable Gray's models based on multiple imputed data (5 imputations were used).

Other potential covariates were excluded for myriad reasons, including substantial proportion of missing values (cold ischemia time, growth failure), collinearity (use of life support at time of transplant), and lack of variation within the cancer subgroup (encephalopathy, spontaneous bacterial peritonitis, portal hypertensive bleeding).

### 2.3. Models

To assess the covariate effects on posttransplant patient survival for the cancer cohort, we used two models in our analysis: Cox PH model and Gray PC-TVC model. Cox PH model provides the estimated average effects while Gray PC-TVC model provides the estimated temporal effects for the covariates of interest. Detailed specifications of these two models are described below.

#### 2.3.1. Cox Proportional Hazards Model

First, we used the Cox PH model, a semiparametric model commonly used in survival analysis. By assuming that the effect of a covariate is multiplicative with respect to the hazard rate and is constant over time, the model is of the form

(1) $$\begin{array}{c} h\left(t\right)=h_{0}\left(t\;|\;X\right)\exp\left(\beta^{\prime }X\right), \end{array}$$

where *h*_0_(*t*) is an unspecified baseline hazard function at time *t*, **X** is a vector of covariates, and **β** is the same dimensional vector of unknown covariate coefficients. 

The coefficients are estimated by maximizing the log partial likelihood function

(2) ℓ(β) =∑i=1nlog(∏j∈Djexp(β′Xj)        ×[∏r=1di{∑j∈Riexp(β′Xj)              −r−1dj∑j∈Diexp(β′Xj)}]−1),

where *i* denotes *n* distinct event times; **X**_*i*_ is the covariate vector of the individual who experienced the event at *t*_*i*_; *R*_*i*_ is the risk set at time *t*_*i*_; *D*_*i*_ is the event set at time *t*_*i*_; and *d*_*i*_ is the number of events occurred at time *t*_*i*_. Here we used the Efron method [5] to adjudicate tied failure times. Note that the model implies the property of proportional hazards, which needs to be tested.

#### 2.3.2. Gray Piecewise Constant Time-Varying Coefficients Model

Gray PC-TVC model is an extension of the Cox PH model. By using a penalized smoothing spline function, Gray PC-TVC model can be used to examine the proportional hazards assumption and to estimate time-varying covariate effects for right-censored data. The model specifies the hazards with the form

(3) $$\begin{array}{c} h\left(t\right)=h_{0}\left(t\;|\;X\right)\exp\left(\beta {\left(t\right)}^{\prime }X\right), \end{array}$$

where **β**_*j*_(*t*) = ∑_*k*_ *α*_*jk*_ *B*_*jk*_(*t*) is the *j*th element of **β**(*t*) and this spline function represents the time-varying coefficient of the *j*th covariate; *B*_*jk*_(*t*), *k* = 1, …,  *K* is a set of *B*-spline basis functions; *α*_*jk*_ is the corresponding basis coefficient. The *B*-spline basis functions are determined by the number of knots and their locations. Knot locations are usually chosen at the times of failure and with roughly equal amounts of failures in between two knots. Under Gray PC-TVC model, the time-varying coefficients are assumed to be constant in between two knots; that is, **β**_*j*_(*t*) is constant for *t* ∈ [*τ*_*k*_, *τ*_*k*+1_) where *τ*_*k*_ is the *k*th knot, *τ*_1_ = 0, and *τ*_*K*+1_ = *T* represents the maximum observed time of failure. The right-continuous step functions of time with jumps may occur at any internal knots [6]. 

To estimate the unknown parameters, a penalty function is added to the log partial likelihood function to prevent overfitting of the data. As for cubic splines, the penalty function has the form

(4) $$\begin{array}{c} \frac{1}{2}\lambda_{j}\int {\left[\beta_{j}^{\prime \prime }\left(s\right)\right]}^{2}ds, \end{array}$$

where *λ*_*j*_ is the smoothing parameter indicating the smoothness and **β**_*j*_′′(*s*) is the second derivative of **β**_*j*_(*s*). The penalty function helps to control the smoothness of the fitted curve through *λ*_*j*_. When *λ*_*j*_ reduces to zero, there is no penalty applied. The larger the *λ*_*j*_, the smoother the curve. The smoothing parameters are usually solved by specifying degrees of freedom. Cubic spline functions tend to be unstable in the right tail of distribution when right censoring yields sparse failure times [4, 7]. In addition to cubic splines, quadratic splines and piecewise constant spline functions can also be applied. The piecewise constant function has the penalty with the form

(5) $$\begin{array}{c} \frac{1}{2}\lambda_{j}\sum_{k=2}^{K+1}{\left(\alpha_{jk}-\alpha_{j,k-1}\right)}^{2}. \end{array}$$

The basis parameters *α* are estimated by maximizing the penalized log partial likelihood function

(6) $$\begin{array}{c} \ell_{p}\left(\alpha \right)=\ell \left(\alpha \right)-\frac{1}{2}\lambda_{j}\sum_{k=2}^{K+1}{\left(\alpha_{jk}-\alpha_{j,k-1}\right)}^{2}, \end{array}$$

where *ℓ*(*α*) is the standard log partial likelihood of Cox model. The penalty function shrinks the size of the jumps at each internal knot in the step functions.

There are two hypotheses of interest: the hypothesis that the *j*th covariate has no overall effect (*H*_0_:  *α*_*jk*_ = 0 for all knots *k*) and the hypothesis that the *j*th covariate satisfies the condition of proportional hazards (*H*_0_: *α*_*jk*_ = *α*_*j*1_ where *α*_*j*1_ *B*_*j*1_ is a linear term in the *j*th covariate coefficient).

There are several conventional methods to check the proportional hazards assumption. For instance, we can create time-covariate interactions and include them in the model with other covariates. Alternatively, we can use graphic methods such as checking the Schoenfeld residual plot. Gray PC-TVC model offers a method of checking the PH assumption by testing whether all piecewise constant coefficients are the same throughout the follow-up time period. It is worth noting that the order and knots of penalized spline functions can be changed based on the characteristics of the data to suit different conditions. After variable selection, a mixed effect analysis can be accomplished by specifying time-independent variables and time-varying variables. The advantage of Gray PC-TVC model is its flexibility on estimating covariate effects, because it can directly capture the temporal changes of covariates when the assumption of proportional hazards is not satisfied. 

### 2.4. Statistical Analysis

The outcome is posttransplant survival, measured from time of transplantation to death. Recipients who were retransplanted, truncated due to administrative censoring, or lost to followup were subject to right censoring in the analysis. 

The selection of explanatory variables in predicting posttransplant survival consists of two steps, univariable selection and multivariable selection. In the univariable selection, each potential covariate specified in the list above was individually fitted using Gray PC-TVC model with 4 degrees of freedom. The number of degrees of freedom used was suggested by Gray [4]. Dummy variables were created at each level of the categorical variables (recipient blood type, donor race/ethnicity and blood type, allocation type, and transplant center location) except for the reference category. Variables with significance at the level of 0.15 were then fitted into the multivariable Gray PC-TVC model to obtain final set of predictors using the forward selection with entry *P* value less than or equal to 0.05. We used the same final set of covariates to refit the data using Cox PH model. All data management and data analyses were implemented in SAS version 9.2 (SAS Institute, Cary, NC, USA) and R version 2.10.0. The Gray PC-TVC models were fit using package *coxspline* (http://cran.r-project.org/) in R. 

## 3. Results

The descriptive statistics for the covariates considered in the univariable models are presented in Table 1. These statistics are shown for all transplant recipients (*n* = 288) and are also broken down by patients who were alive (*n* = 237) and those who died (*n* = 51) during the posttransplant follow-up period. Median follow-up time of all recipients was 612.5 days (1.68 years).

In the overall sample of 288 recipients, there were 11 recipients with blood type AB, only one of whom died. The number of days of posttransplant survival and the vital status for these 11 recipients are provided in Appendix A. Kaplan-Meier survival estimates of the posttransplant survival time between those with blood type AB and those with other blood types (Appendix A) show that recipients with other blood types died faster compared to those with blood type AB. For blood type AB group, the only jump on the survival function occurred at follow-up day 616 when the recipient died after two subjects were right-censored. The two survival curves do not cross, and the recipients with blood type AB seem to have higher overall survival than that of those with other blood types, but the difference between the two curves is not significant (logrank test *P* = 0.336). 

We also estimated Kaplan-Meier survival curves by donor blood type (Appendix B) and found that survival curves for the four blood types (A, B, AB, and O) were not parallel. The Tarone-Ware test indicates a significant difference of posttransplant survival rates among donor blood types (Tarone-Ware Chi-square statistic = 8.0053 with 3 degrees of freedom, *P* = 0.046), with donor blood type O having a lower posttransplant survival rate than other donor blood types.

 Similarly, only 16 recipients used ventilator at the time of transplant and 6 (37.5%) of them died. Given the details in Appendix C, the overall survival for recipients who used ventilator at time of transplant is lower than those who did not (Tarone-Ware test *P* = 0.001). This indicates that the recipients who used ventilator are transplanted with a worse health condition than those who did not and are unlikely to survive for a long period.

After fitting univariable Gray PC-TVC models, covariates that were statistically significant at the level of 0.15 included recipient characteristics (age, female gender, race (recoded as black versus non-black); laboratory values (albumin, bilirubin, creatinine) at time of transplant; positive CMV; use of a ventilator at time of transplant; presence of ascites at transplant); donor characteristics (age, blood type, race/ethnicity); and recipient-donor blood type compatibility. 

Based on these results from the univariable models, significant covariates were then included in the forward selection procedure with entry *P*-value of 0.05 to obtain the final multivariable Gray PC-TVC model. Starting with the most significant, explanatory variables were sequentially added to the model until none of the remaining variables was significant (*P* < 0.05). The final multivariable model included 5 covariates: donor blood type, recipient creatinine at time of transplant, use of a ventilator at time of transplant, positive CMV, and recipient gender. We checked the two-way interactions for the final multivariable models, but none of the interaction terms was significant at level of 0.05. We then performed a Cox PH model with these 5 variables.

Table 2 summarizes the estimation and hypothesis testing results for both of these models. Beginning with the Cox PH model, the table presents the estimated average coefficients (log hazard ratio) with 95% confidence intervals, as well as *P*-values of testing the significance of the average effect. Based on these results, only creatinine (*P* = 0.001) and positive CMV (*P* = 0.023) had significant average effects on posttransplant survival. Recipients with higher creatinine levels had increased risk of death; likewise, recipients with positive CMV had higher risks of death than those testing negative. 

The remainder of the table presents results from the Gray PC-TVC model, with *P-*values indicating (1) the overall effect of the covariate on posttransplant survival and (2) whether the covariate violates the proportional hazards assumption. As noted above, all five variables significantly affect survival and were therefore retained in the final model. In addition, the *P*-values associated with the proportional hazards assumptions indicated that donor blood type AB, use of a ventilator, and female gender violate the proportional hazards assumption, indicating that the effects are not constant over time. Therefore Cox PH model may not be sufficient to capture the temporal changes of these covariate effects.

**Figure 1:**
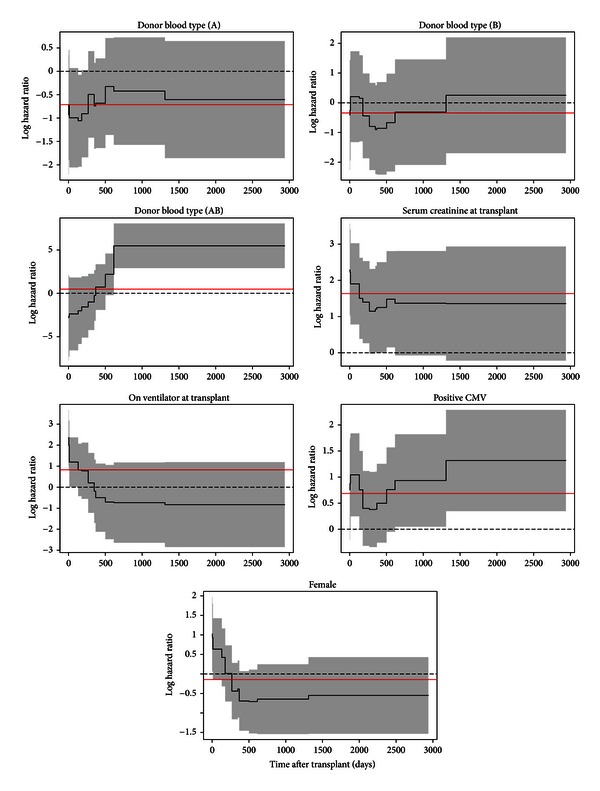
Time-varying covariate effects (black solid lines) with 95% confidence intervals (shaded areas) are from the final Gray PC-TVC model with 4 degrees of freedom for each variable. The constant covariate effects (red solid lines) are estimated from the Cox PH model.

Figure 1 depicts changes in the covariate effects over time based on the final multivariable Gray PC-TVC model. The covariate effects in black solid lines are from Gray PC-TVC model with 4 degrees of freedom for each variable, with pointwise 95% confidence intervals shaded in grey. For comparison, the estimated average covariate effects from Cox PH model are shown in red.

The most notable effect is that recipients who received livers from donors of blood type AB tend to have lower risks of death in the first two years, but then experience significantly higher risks afterwards. The Gray PC-TVC model shows that donor blood type AB has a strong nonproportional effect on posttransplant survival for recipients diagnosed with cancer, a finding that cannot be observed at all in the Cox PH model. (It is unclear why recipients who received livers from donors with AB blood type had better survival. The most obvious hypothesis is that this finding pointed to the benefit of exact matches between recipient and donor blood types. Unfortunately, the data do not support this hypothesis). 

Ventilator use at time of transplant results in a strong decreasing trend. In the short term, patients who required ventilator support at time of transplant are sicker and have higher risks of death than those who do not, but the difference diminishes over time and these patients have better long-term survival. In contrast, results of the Cox PH model suggest that ventilator use is not significant with average hazard ratio of 2.3 (e^0.83^). Similarly, female recipients tend to have higher risk at the beginning, but better survival on the long run than male recipients. The effect of gender is marginally significant in the Gray PC-TVC model, but trivial in the Cox PH model. For other covariates like creatinine at time of transplant and positive CMV, Cox and Gray both report significance, but none of the effects varies over time. For these variables, as the proportional hazards assumption is not violated, it may be reasonable to use the Cox PH model. It is therefore of interest to consider an additional model allowing for both time-varying and time-invariant covariate effects. This was accomplished by a Gray PC-TVC model combining both linear terms and spline functions of covariates. Based on the previous results, we fit donor blood type, recipient gender, and ventilator usage as spline-based time-varying effect functions and positive CMV and serum creatinine as linear fixed effect covariates. The results are shown in Table 3. Compared with the results of our previous fitted Gray model (all covariate effects are time-varying), the estimated time-varying effects of covariates in the additional model are very similar. In addition, the estimated time-invariant effects of covariates also show similar magnitude as the coefficients obtained from the previous Cox model (all covariates are time-invariant). 

**Figure 2:**
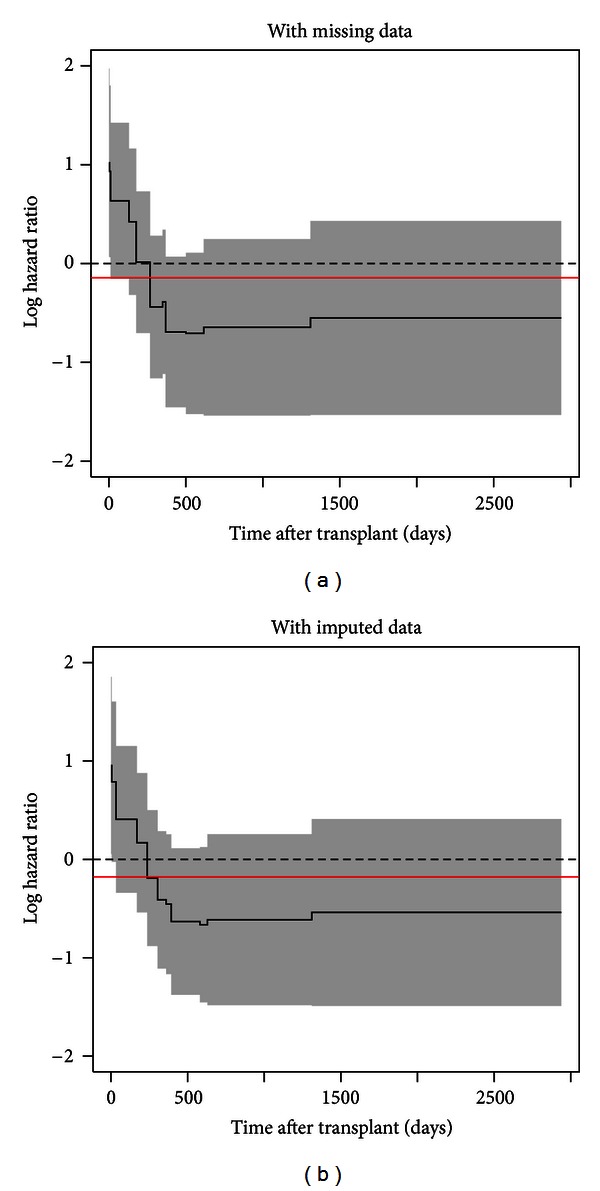
Time-varying covariate effects of recipient gender from the data with missing values (a) and with multiple imputation (b). The constant covariate effects (red solid lines) are estimated from each Cox PH model.

Finally, we reran the multivariable Gray model with the same final set of covariates as in complete-case analysis based on the multiple imputed data. The results are shown in Table 4. Compared to the results in Table 2 (assumed MCAR), the magnitude and functional trend of estimated coefficients in Table 4 do not change noticeably. However, the effect of recipient gender becomes nonsignificant from *P* = 0.045 to 0.084.  Figure 2 depicts the estimated covariate effects for gender when missing data was treated as MCAR Figure 2(a) and treated as MAR, Figure 2(b). Although *P*-value changes, the difference in patterns of the time-varying effects is not detectable. 

## 4. Discussion 

Liver transplant survival analyses often apply standard Cox PH model for estimation. In the present paper, we applied the Gray penalized spline method as an alternative to the Cox PH model to determine the important predictors and estimate their time-varying effects. The time-invariant effects were estimated as well using standard Cox PH model on the same set of predictors. We included only the pediatric patients with a diagnosis of cancer (primary liver malignancy) from the UNOS data. The results were reported graphically and numerically, showing key differences between two models. Donor AB blood type, ventilator use, and recipient gender are significant in Gray PC-TVC model but not significant in Cox PH model. Furthermore, their effects vary over time after transplant. Although Cox PH model provided average estimates of coefficients, it failed to capture changes during observation period.

**Figure 3:**
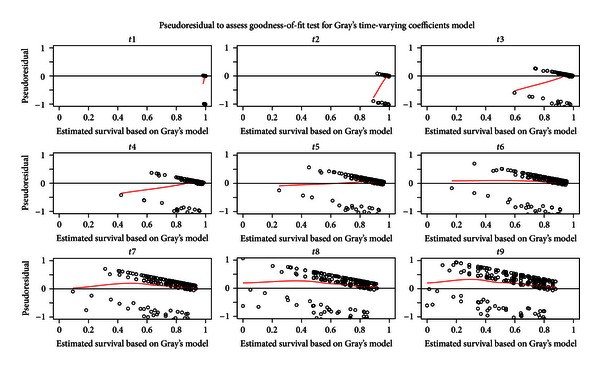
Goodness-of-fit test for Gray PC-TVC model using pseudoresiduals (dotted points) and lowess smoothed curves (red solid lines) against the estimated survival rates at each of the preselected time points.

**Figure 4:**
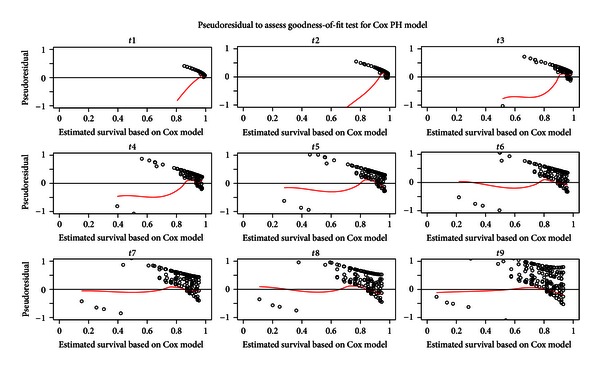
Goodness-of-fit test for Cox PH model using pseudoresiduals (dotted points) and lowess smoothed curves (red solid lines) against the estimated survival rates at each of the preselected time points.

While the multiplicative hazards assumption holds without specifying the parametric form of baseline hazard function, Gray PC-TVC model has greater flexibility when the regression covariate effects change over time. It also can be used to check the proportional hazards assumption for the data. Many methods can be used to check the adequacy of a Cox model; however, no methods published can be used to check the overall goodness-of-fit for a Gray PC-TVC model. Based on the method proposed by Kang [8], the graphical check of goodness-of-fit for the final Gray PC-TVC model and Cox PH model using pseudoresiduals is presented in Figures 3 and 4, respectively. In the figures, the pseudoresiduals were calculated and plotted along with the lowess smoothed curves against the estimated survival rates at each of the nine preselected time points. Since the lowess smoothed curves of pseudoresiduals in Figure 3 stay around zero and are stable at most of time points (except time points 2 and 3) without any significant departure or tendency, we can conclude that the final multivariable Gray model shows a good fit in estimating posttransplant survival function. In Figure 4, however, the pseudoresiduals clearly illustrate departure from zero and some tendency. Therefore, final multivariable Cox model shows lack of fit to the data. 

The main limitation of this analysis is the small sample size, which limits the generalizability of our findings and the set of covariates that could be considered. To examine the stability of the results given the uneven distribution of donor blood type and small sample sizes, we conducted sensitivity analysis. We first removed two observations with donor blood type AB and refit the final multivariable Gray PC-TVC and Cox PH models. The results showed that after removing these two observations, recipient gender becomes nonsignificant from *P* = 0.045 to *P* = 0.057. After checking the time-varying pattern of the recipient gender effects (Appendix D), the effects are very similar and do not show noticeable difference. 

However, transplant recipients with liver cancer were an appropriate cohort for meeting the primary goal of this paper, comparing Cox PH and Gray PC-TVC models and demonstrating the usefulness of more flexible approaches for estimating survival in some diseases. As the data here illustrated, using a Cox PH model in diseases where the proportional hazards assumptions are not satisfied can potentially lead to incorrect specifications and ignore the effect of important covariates. 

## 5. Conclusions

While Cox PH model provided reasonable average results in estimating covariate effects on posttransplant survival, Gray model with piecewise constant penalized splines showed more details of how the effects change over time. An example of this is the effect of being on a ventilator at time of transplant. Requiring ventilator support indicates significant acute illness, often not directly related to liver disease. It therefore makes sense that the effect of being on a ventilator might dramatically affect early postoperative mortality but that the effect would decline over time as the reason for ventilator support was treated. Because the Cox PH model must average these effects to be constant over time, the higher early mortality “cancels” the lower later mortality and the effect of that variable is not significant in a Cox PH model. 

Choosing the optimal time to perform transplantation is an essential way to improve patient survival. The time-varying coefficients model is more flexible than the traditional Cox PH model to estimate temporal changes that influence timing decisions and predictions about posttransplant survival.

## Figures and Tables

**Figure 5:**
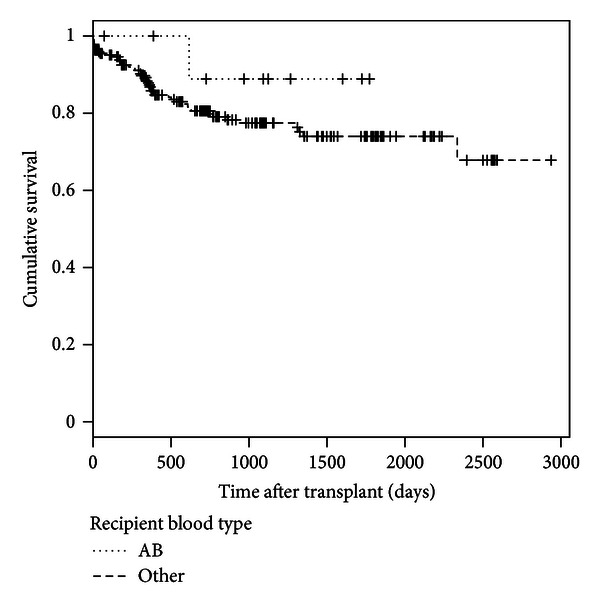
Kaplan-Meier survival estimates of the posttransplant survival time between recipients with blood type AB and those with other blood types (logrank Chi-square statistic = 0.9264, *P* = 0.336).

**Figure 6:**
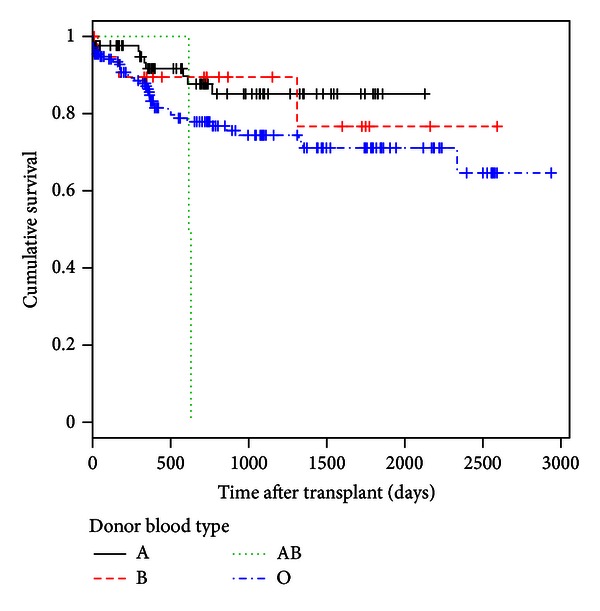
Kaplan-Meier survival estimates of the posttransplant survival by donor blood type (Tarone-Ware Chi-square statistic = 8.0053, *P* = 0.046).

**Figure 7:**
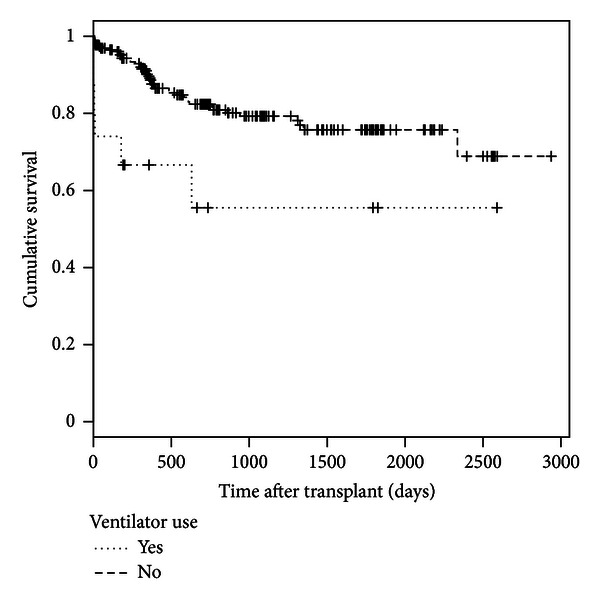
Kaplan-Meier survival estimates of the posttransplant survival time between recipients who used ventilator at time of transplant and those without using ventilator (Tarone-Ware Chi-square statistic = 11.4723, *P* = 0.001).

**Figure 8:**
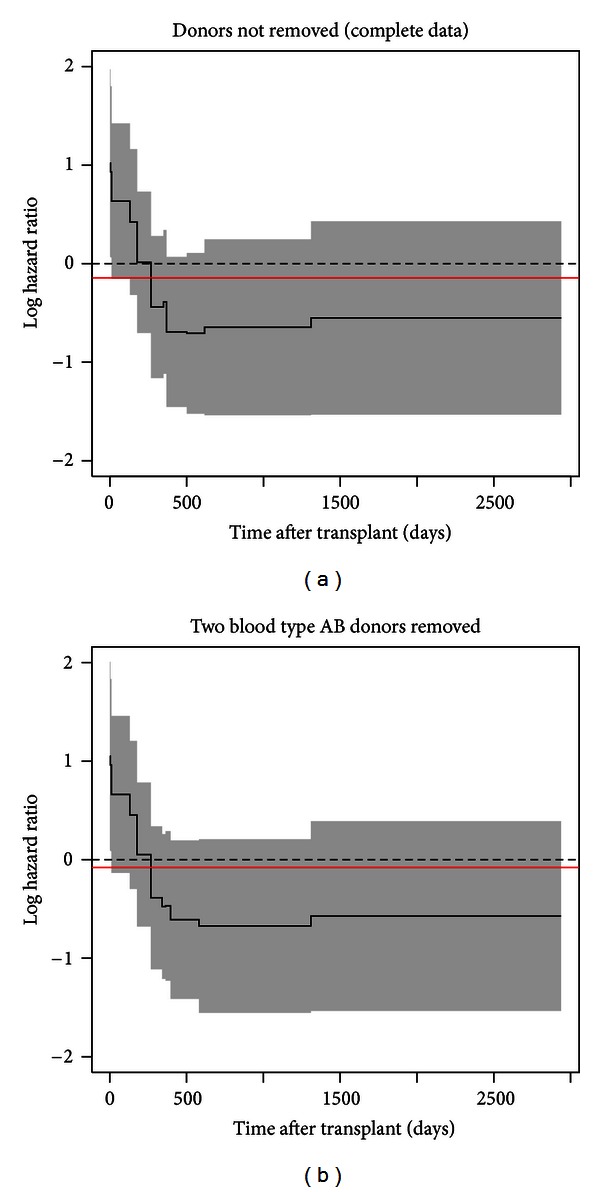


**Table 1:** Characteristics of the covariates considered in the univariable models.

Characteristics	All recipients (*N* = 288)	Patient outcome	
		Alive (*N* = 237)	Died (*N* = 51)
Recipient characteristics			
Demographics			
Age, median, mean ± SD (years)*	2.00, 4.36 ± 4.83	2.00, 4.12 ± 4.82	4.00, 5.51 ± 4.78
Gender, no. (%)			
Female	121 (42.01)	98 (41.35)	23 (45.10)
Male	167 (57.99)	139 (58.65)	28 (54.90)
Race/ethnicity, no. (%)			
Black	24 (8.33)	20 (8.44)	4 (7.84)
Nonblack	264 (91.67)	217 (91.55)	47 (92.16)
Medical/clinical covariates			
Blood type, no. (%)			
A	106 (36.81)	91 (38.40)	15 (29.41)
AB	11 (3.82)	10 (4.22)	1 (1.96)
B	38 (13.19)	32 (13.50)	6 (11.76)
O	133 (46.18)	104 (43.88)	29 (56.86)
On ventilator, no. (%)			
Yes	16 (5.56)	10 (4.22)	6 (11.76)
No	271 (94.10)	226 (95.36)	45 (88.24)
Unknown	1 (0.35)	1 (0.42)	0 (0.00)
Laboratory values, median, mean ± SD			
Albumin (g/dL)	3.80, 3.66 ± 0.74	3.80, 3.70 ± 0.71	3.60, 3.48 ± 0.83
Bilirubin (mg/dL)	0.50, 2.06 ± 5.91	0.40, 1.63 ± 4.53	0.70, 4.11 ± 9.92
Serum creatinine (mg/dL)^†^	0.40, 0.45 ± 0.26	0.40, 0.42 ± 0.23	0.55, 0.59 ± 0.37
INR	1.10, 1.32 ± 0.87	1.10, 1.30 ± 0.91	1.20, 1.39 ± 0.64
Presence of ascites, no. (%)			
Yes	38 (13.19)	27 (11.39)	11 (21.57)
No	166 (57.64)	139 (58.65)	27 (52.94)
Unknown	84 (29.17)	71 (29.96)	13 (25.49)
Presence of portal vein thrombosis, no. (%)			
Yes	13 (4.51)	11 (4.64)	2 (3.92)
No	265 (92.01)	217 (91.56)	48 (94.12)
Unknown	10 (3.47)	9 (3.80)	1 (1.96)
Previous abdominal surgery, no. (%)			
Yes	124 (43.06)	99 (41.77)	25 (49.02)
No	145 (50.35)	123 (51.90)	22 (43.14)
Unknown	19 (6.59)	15 (6.33)	4 (7.84)
Positive cytomegalovirus (CMV) test, no. (%)			
Yes	81 (28.13)	60 (25.32)	21 (41.18)
No	207 (71.88)	177 (74.68)	30 (58.82)
Other characteristics			
Donor type, no. (%)			
Deceased	256 (88.89)	210 (88.61)	46 (90.20)
Living	31 (10.76)	26 (10.97)	5 (9.80)
Unknown	1 (0.35)	1 (0.42)	0 (0.00)
Donor age, median, mean ± SD (years)^‡^	14.00, 15.35 ± 14.53	12.00, 14.40 ± 14.07	17.00, 19.75 ± 15.92
Donor gender, no. (%)			
Female	112 (38.89)	90 (37.97)	22 (43.14)
Male	175 (60.76)	146 (61.60)	29 (56.86)
Unknown	1 (0.35)	1 (0.42)	0 (0.00)
Donor race/ethnicity, no. (%)			
White	152 (52.78)	129 (54.43)	23 (45.10)
Black	51 (17.71)	42 (17.72)	9 (17.65)
Hispanic	70 (24.31)	56 (23.63)	14 (27.47)
Asian	12 (4.17)	7 (2.95)	5 (9.80)
Other	3 (1.04)	3 (1.27)	0 (0.00)
Donor blood type, no. (%)			
A	85 (29.51)	76 (32.07)	9 (17.65)
AB	2 (0.69)	0 (0.00)	2 (3.92)
B	21 (7.29)	18 (7.59)	3 (5.88)
O	179 (62.15)	142 (59.92)	37 (72.55)
Unknown	1 (0.35)	1 (0.42)	
ABO compatible, no. (%)			
Yes	283 (98.26)	233 (98.31)	50 (98.04)
No	4 (1.39)	3 (1.27)	1 (1.96)
Unknown	1 (0.35)	1 (0.42)	0 (0.00)
Transplantation-related characteristics			
Active exception at time of transplant, no. (%)			
Yes	128 (44.44)	109 (45.99)	19 (37.25)
No	160 (55.56)	128 (54.01)	32 (62.75)
Unknown	0 (0.00)	0 (0.00)	0 (0.00)
Transplant year, no. (%)			
2002	16 (5.56)	10 (4.22)	6 (11.76)
2003	18 (6.25)	15 (6.33)	3 (5.88)
2004	41 (14.24)	32 (13.50)	9 (17.65)
2005	41 (14.24)	32 (13.50)	9 (17.65)
2006	35 (12.15)	27 (11.39)	8 (15.69)
2007	33 (11.46)	26 (10.97)	7 (13.73)
2008	47 (16.32)	40 (16.88)	7 (13.73)
2009	32 (11.11)	32 (13.50)	0 (0.00)
2010	24 (8.33)	22 (9.28)	2 (3.92)
Unknown	1 (0.35)	1 (0.42)	0 (0.00)
Center location (region), no. (%)			
(1) CT, ME, MA, NH, RI	12 (4.17)	10 (4.22)	2 (3.92)
(2) DC, DE, MD, NJ, PA, WV	49 (17.01)	40 (16.88)	9 (17.65)
(3) AL, AR, FL, GA, LA, MS, PR	27 (9.38)	22 (9.28)	5 (9.80)
(4) OK, TX	20 (6.94)	15 (6.33)	5 (9.80)
(5) AZ, CA, NV, NM, UT	75 (26.04)	66 (27.85)	9 (17.65)
(6) AK, HI, ID, MT, OR, WA	8 (2.78)	6 (2.53)	2 (3.92)
(7) IL, MN, ND, SD, WI	25 (8.68)	18 (7.59)	7 (13.73)
(8) CO, IA, KS, MO, NE, WY	20 (6.94)	16 (6.75)	4 (7.842)
(9) NY, VT	14 (4.86)	12 (5.06)	2 (3.92)
(10) IN, MI, OH	27 (9.38)	23 (9.70)	4 (7.84)
(11) KY, NC, SC, TN, VA	11 (3.82)	9 (3.80)	2 (3.92)
Allocation type, no. (%)			
Local	134 (46.53)	106 (44.73)	28 (54.90)
Regional	106 (36.81)	87 (36.71)	19 (37.25)
Other	47 (16.32)	43 (18.14)	4 (7.84)
Unknown	1 (0.35)	1 (0.42)	0 (0.00)
Procurement distance, median, mean ± SD (miles)^§^	156.00, 300.36 ± 411.36	169.00, 313.10 ± 421.48	89.00, 241.43 ± 358.70
Partial or split donor organ, no. (%)			
Partial or split	109 (37.85)	88 (37.13)	21 (41.18)
Whole	178 (61.81)	148 (62.45)	30 (58.82)
Unknown	1 (0.35)	1 (0.42)	0 (0.00)
Waiting time, median, mean ± SD (days)	29.00, 45.35 ± 74.38	28.00, 48.00 ± 80.87	30.00, 33.04 ± 26.42

SD: standard deviation.

*The age at time of transplant of one child (alive) was missing.

^†^Serum creatinine values were missing for 18 children: 15 alive and 3 dead.

^‡^The age of one donor was missing.

^§^Procurement distance values were missing for one child (alive).

**Table 2:** Estimated log hazard ratios for testing overall covariate effects and the test results of nonproportionality (nonprop) using Cox proportional hazards (PH) model and Gray piecewise constant time-varying coefficients (PC-TVC) model.

Covariate	Cox PH		Gray PC-TVC	
	Log hazard ratio (95% CI)	*P* value	*P* value	Nonprop*P value
Donor blood type				
A	−0.711 (−1.484, 0.062)	0.071	0.163	0.498
B	−0.343 (−1.534, 0.847)	0.572	0.679	0.480
AB	0.482 (−1.208, 2.171)	0.576	0.001	0.001
Serum creatinine (mg/dL)	1.635 (0.695, 2.575)	0.001	0.001	0.527
On ventilator	0.828 (−0.166, 1.821)	0.102	0.002	0.012
Positive CMV	0.686 (0.096, 1.275)	0.023	0.017	0.163
Female gender	−0.143 (−0.731, 0.445)	0.633	0.045	0.007

*Null hypothesis: the proportional hazards (PH) assumption is not violated.

**Table 3:** Estimated time-varying and time-invariant coefficients and *P* values for testing overall covariate effects and proportional hazards.

Covariate	Log hazard ratio		Overall *P* value	Nonprop *P* value
	Min	Max		
Donor blood type				
A	−1.081	−0.333	0.140	0.481
B	−0.900	0.208	0.700	0.510
AB	−2.741	5.370	0.001	0.001
Serum creatinine (mg/dL)	1.622		<0.001	
On ventilator	−0.844	2.364	0.002	0.011
Positive CMV	0.697		0.010	
Female gender	−0.738	1.013	0.040	0.006

**Table 4:** Estimated coefficients and test *P* values using multiple imputed data.

Covariate	Cox PH		Gray PC-TVC	
	Log hazard ratio (95% CI)	*P* value	*P* value	Nonprop *P* value
Donor blood type				
A	−0.608 (−1.341, 0.126)	0.104	0.234	0.547
B	−0.387 (−1.577, 0.803)	0.524	0.694	0.533
AB	0.486 (−1.205, 2.176)	0.573	0.001	0.003
Serum creatinine (mg/dL)	1.698 (0.768, 2.626)	<0.001	<0.001	0.468
On ventilator	0.842 (−0.149, 1.834)	0.096	0.003	0.016
Positive CMV	0.670 (0.098, 1.243)	0.022	0.014	0.134
Female gender	−0.178 (−0.750, 0.394)	0.542	0.084	0.018

**Table 5:** Numbers of days of posttransplant survival for the 11 recipients with blood type AB.

Survival time	616	1124	387	968	726	1724	1600	1266	1092	1773	72
Died	1	0	0	0	0	0	0	0	0	0	0

**Table 6:** Results of sensitivity analysis.

Covariate	Gray model A*		Gray model B**	
	Overall *P* value	Nonprop *P* value	Overall *P* value	Nonprop *P* value
Donor blood type				
A	0.163	0.498	0.143	0.394
B	0.679	0.480	0.695	0.500
AB	0.001	0.001		
Serum creatinine (mg/dL)	0.001	0.527	0.001	0.545
On ventilator	0.002	0.012	0.002	0.034
Positive CMV	0.017	0.163	0.020	0.210
Female gender	0.045	0.007	0.057	0.010

*Previous final multivariable Gray model.

**Gray model with two observations removed.

## Appendices

### A. The Posttransplant Survival Functions for Recipients with Blood Type AB

See Table 5 and Figure 5.

### B. The Posttransplant Survival Functions by Donor Blood Type

See Figure 6.

### C. The Posttransplant Survival Functions for Ventilator Users and Nonusers at Time of Transplant

See Figure 7.

### D. Sensitivity Analysis for the Impact of Extreme Distribution of Donor Blood Type on Covariate Effects

See Table 6 and Figure 8.
